# The implementation of a noninvasive lymph node staging (NILS) preoperative prediction model is cost effective in primary breast cancer

**DOI:** 10.1007/s10549-022-06636-x

**Published:** 2022-07-05

**Authors:** Ida Skarping, Kristoffer Nilsson, Looket Dihge, Adam Fridhammar, Mattias Ohlsson, Linnea Huss, Pär-Ola Bendahl, Katarina Steen Carlsson, Lisa Rydén

**Affiliations:** 1 Division of Oncology, Department of Clinical Sciences, Lund University, Lund, Sweden; 2 Department of Clinical Physiology and Nuclear Medicine, Skåne University Hospital, Lund, Sweden; 3 The Swedish Institute for Health Economics, Lund, Sweden; 4 Division of Surgery, Department of Clinical Sciences, Lund University, Lund, Sweden; 5 Department of Plastic and Reconstructive Surgery, Skåne University Hospital, Malmö, Sweden; 6 Division of Computational Biology and Biological Physics, Department of Astronomy and Theoretical Physics, Lund University, Lund, Sweden; 7 Division of Surgery, Department of Clinical Sciences Helsingborg, Lund University, Lund, Sweden; 8 Department of Surgery, Helsingborg General Hospital, Helsingborg, Sweden; 9 Department of Clinical Sciences, Health Economics, Lund University, Malmö, Lund, Sweden; 10 Department of Surgery and Gastroenterology, Skåne University Hospital, Malmö, Sweden

**Keywords:** Breast neoplasm, Artificial neural network, Staging, Axillary lymph nodes, Cost-effectiveness

## Abstract

**Purpose:**

The need for sentinel lymph node biopsy (SLNB) in clinically node-negative (cN0) patients is currently questioned. Our objective was to investigate the cost-effectiveness of a preoperative noninvasive lymph node staging (NILS) model (an artificial neural network model) for predicting pathological nodal status in patients with cN0 breast cancer (BC).

**Methods:**

A health-economic decision-analytic model was developed to evaluate the utility of the NILS model in reducing the proportion of cN0 patients with low predicted risk undergoing SLNB. The model used information from a national registry and published studies, and three sensitivity/specificity scenarios of the NILS model were evaluated. Subgroup analysis explored the outcomes of breast-conserving surgery (BCS) or mastectomy. The results are presented as cost (€) and quality-adjusted life years (QALYs) per 1000 patients.

**Results:**

All three scenarios of the NILS model reduced total costs (–€93,244 to –€398,941 per 1000 patients). The overall health benefit allowing for the impact of SLNB complications was a net health gain (7.0–26.9 QALYs per 1000 patients). Sensitivity analyses disregarding reduced quality of life from lymphedema showed a small loss in total health benefits (0.4–4.0 QALYs per 1000 patients) because of the reduction in total life years (0.6–6.5 life years per 1000 patients) after reduced adjuvant treatment. Subgroup analyses showed greater cost reductions and QALY gains in patients undergoing BCS.

**Conclusion:**

Implementing the NILS model to identify patients with low risk for nodal metastases was associated with substantial cost reductions and likely overall health gains, especially in patients undergoing BCS.

**Supplementary Information:**

The online version contains supplementary material available at 10.1007/s10549-022-06636-x.

## Introduction

Most breast cancer (BC) patients present with local disease in the breast without distant metastases at diagnosis. Routinely, clinical and radiological assessments of axillary lymph node (ALN) status are performed. Since the negative predictive value of imaging, most commonly axillary ultrasound, is considered insufficient [1, 2] in patients with negative findings, ALN staging via sentinel lymph node (SLN) biopsy (SLNB) is recommended for all clinically node-negative (cN0) patients [3, 4]. However, for most BC patients, SLNB will result in benign findings, and therefore, the SLNB was only diagnostic and not therapeutic.

Although SLNB is the standard of care, it is not perfect and has a well-established false-negative rate of 10% [5], implicating that the SLNB technique is associated with a risk of leaving metastatic nodes behind. Although SLNB is a minor surgical procedure, especially in comparison to axillary lymph node dissection (ALND), it is still associated with both short- and long-term side effects along with high health care costs. Postoperative swelling, paresthesia, and symptoms associated with arm morbidity, i.e., lymphedema and symptoms of arm swelling, are reported complications [6, 7].

In recent years, the role of axillary surgery, including SLNB, has been explored and questioned. In patients with cN0 BC and 1–2 positive SLNs (including macrometastases > 2 mm), the presented results from the Z0011 trial showed no superior survival for patients treated with SLNB alone compared with patients undergoing ALND, supporting the use of only SLNB in patients with 1–2 metastatic lymph nodes who receive breast-conserving surgery (BCS) and postoperative adjuvant therapy [8]. Moreover, although SLNB provides staging of the axilla, molecular biology is more informative and indicative for the choice of systemic treatment [9]. As a consequence, according to recent ASCO guidelines, SLNB is not recommended for a patient of higher age (≥ 70 years) presenting with cN0, hormone receptor (HR)-positive, and human epidermal growth factor receptor 2 (HER2)-negative BC if the patient will receive adjuvant endocrine treatment [3, 4].

There is a clear trend of management of the axilla in BC, tending toward minimizing axillary surgery and even omitting SLNB. The SOUND trial [10], which randomized early BC patients (T1cN0) to receive either SLNB or observation alone, may prove that performing SLNB in low-risk BC patients is futile. In both the INSEMA trial [9] and the BOOG 2013–08 trial [11], BC patients undergoing BCS (T1-2cN0) were randomized to receive or not receive SLNB. The results of these trials are highly anticipated.

Better tools for predicting the SLN status in cN0 BC could reduce the occurrence of unbeneficial SLNBs in patients with a low risk of having any SLN metastasis. We developed an artificial neural network (ANN) model, the noninvasive lymph node staging (NILS) prediction model, producing a probability score of a patient being node negative [12]. A validation study is currently being conducted (ISRCTN14341750), and the study protocol has been published [13]. Before broadly implementing new health care technologies and decision tools such as the NILS model, careful considerations of costs and health consequences are needed in addition to information on efficacy and safety [14, 15].

Our aim was to investigate the cost-effectiveness of implementing the NILS model for predicting pathological nodal status (pN) in patients with cN0 BC stratified by mastectomy and BCS.

## Methods

### The NILS prediction model

The model is based on ten clinicopathological parameters of which age and eight other parameters (tumor size, multifocality, estrogen receptor status, histological type, progesterone receptor status, mode of detection, tumor localization in the breast, and Ki-67 positivity) are easily accessible from mammograms and core needle biopsies for the prediction of nodal status in cN0 BC patients. The 10th parameter, vascular invasion, was included in the original NILS model and can be predicted by the other included variables [12].

### Health economic model

We developed a health economic model to analyze the cost-effectiveness of the NILS model in patients with cN0 BC without preoperative chemotherapy. The population was divided into groups with positive and negative pathological nodal statuses (pN + and pN0), in which pN + BC was defined as BC with micrometastases or macrometastases, and pN0 BC included N0 (i +) BC, i.e., isolated tumor cells [16]. According to The South Sweden Regional Tumor Registry, 32% of women with cN0 BC have pN + BC. This proportion was used in the model [17].

**Fig. 1:**
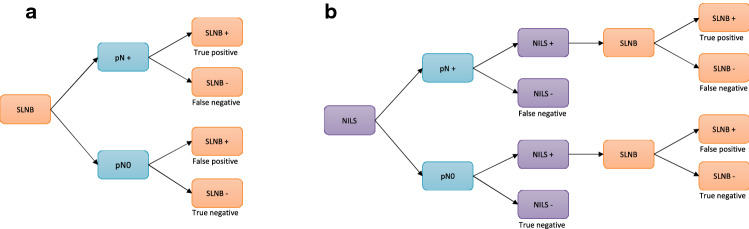
Decision tree model: **A** Standard of care using SLNB as the staging procedure, as represented by the decision tree model. **B** Using the NILS prediction model to predict nodal status, where SLNB will only be performed after a positive NILS prediction model result, as represented by the decision tree model

The standard of care staging procedure based on SLNB is shown in Fig. 1A. The sensitivity and specificity of SLNB to detect pN + and pN0 BC were used to calculate the proportions of true positive, false negative, false positive, and true negative diagnoses. The model used a sensitivity of 92.3% based on a Swedish multicenter study of SLNB in cN0 BC patients [5] and a specificity of 100% (Table S1).

The diagnostic process for the NILS model, in which SLNB is only performed after N + disease is predicted from the NILS model, is shown in Fig. 1B. Here, the sensitivity and specificity for both the NILS model and SLNB were used to calculate the proportions of positive and negative diagnoses. Three scenarios for the accuracy of the original NILS model for the prediction of lymph node status (N0 versus N +) were analyzed based on the results from Dihge et al. [12]. Three cutoffs for classification of ALN status (N0 versus N +) were set in the published original NILS model [12]: 1) based on maximized negative predictive value aimed at identifying individuals with a very low probability of axillary disease and 2–3) based on the accepted FNR (5–10%) for the SLNB technique.

- Scenario 1: 99% sensitivity and 11% specificity
- Scenario 2: 95% sensitivity and 25% specificity
- Scenario 3: 90% sensitivity and 37% specificity

The NILS model was developed to identify individuals with a very low probability of axillary disease, and the model displayed high sensitivity but low specificity. Thus, many patients with benign nodal status (pN0) would be subjected to further standard SLNB. Although not ideal, the current classification indicates appropriate cautiousness of the current model.

### Treatments

All patients diagnosed with pN0 BC (true negative and false negative) were assumed to follow the same treatment program. Radiotherapy after BCS and HER2-targeted therapy was assumed to be administered regardless of ALN status. On the other hand, receipt of ALND, radiation after mastectomy, extended hormonal therapy beyond 5 years, and adjuvant chemotherapy was assumed to be affected by the ALN status. All patients diagnosed with pN + BC were assumed to undergo subsequent ALND after SLNB, in line with current national guidelines [16]. The model includes the risks of lymphedema, seroma, and infection as complications following SLNB and ALND (Table S1). We used the following rates of lymphedema in the model: 0.4% without axillary surgery, 6.3% after SLNB, and 22.3% after SLND and ALND [18, 19].

### Survival and recurrence

The yearly BC mortality and recurrence rate were based on 10-year survival and recurrence data from the Swedish Multicenter Cohort Study [20]. Increased mortality and recurrence rates for false negatives due to omitted radiotherapy, chemotherapy, and extended hormonal treatment (ER + BC) were derived from EBCTCG meta-analyses [21–23]. BC mortality and recurrence were calculated over 10 years in the model. Age-adjusted all-cause mortality based on life table data from Statistics Sweden was included in a life-long perspective in the model.

### Health care costs

The health economic model included costs of surgery, adjuvant treatments, recurrence, metastatic disease, and complications related to SLNB and ALND (Table S1). The surgical costs were calculated from data from a series of 1405 BC surgeries performed during 2018–2020 in Region Skåne, Sweden. No extra costs from the use of routinely collected data from mammography and core needle biopsy reports were considered. No cost for using the NILS model was included in the analysis.

### Quality of life

Health effects are measured in quality-adjusted life years (QALYs), a measurement of effect that combines both the length of life and the quality of life (QoL). All included QoL data were measured by the generic QoL instrument EQ-5D [24]. Age-adjusted quality of life weights for the Swedish general population were used [25]. The analysis included QoL decrements for adjuvant chemotherapy, recurrence of BC [26], and lymphedema (Table S1). There is uncertainty regarding the impact of lymphedema on QoL. One study reported a decrease of 0.1 [27], while another study found no impact on QoL [28]. The results are therefore presented both with and without a life-long QoL decrement of 0.1 for lymphedema. An additional analysis investigated the minimum QoL decrement for lymphedema at which the NILS model would lead to gains in total QALYs in all three scenarios compared with standard of care.

### Cost-effectiveness

Cost-effectiveness was measured by incremental changes in QALYs and health care costs. An intervention was dominant if it led to both health gains and cost reductions. All costs and health gains were discounted with an annual discount rate of 3% in accordance with Swedish national guidelines [29].

## Results

Compared to standard of care (i.e., SLNB for all cN0 patients), using the NILS model for prediction of ALN status reduced the number of SLNBs, and the difference was higher with lower sensitivity and higher specificity of the NILS model (i.e., as represented by Scenarios 1 to 3); SLNB was omitted for 78 (7.8%) and 284 (28%) of 1000 patients in Scenarios 1 and 3, respectively (Table 1). The reduction of SLNB based on the NILS model was associated with a reduction in total costs compared to the standard of care of € 93,244 (Scenario 1) to € 398,941 (Scenario 3) per 1000 patients, primarily due to lower costs of surgery (SLNB and subsequent ALND) and associated complications but also as a result of an expected reduction in the use of adjuvant therapies (Table 2). However, implementation of the NILS model also led to lost life years (0.6 to 6.5 life years per 1000 patients) and 0.1–0.6 more BC-related deaths than the standard of care due to a somewhat higher risk of undetected pN + BC resulting in more recurrences and metastatic disease. When the life-long lymphedema QoL decrement was set to 0.1, the intervention led to a gain in total QALYs (7.0 to 26.9 QALYs) (Table 1). In contrast, when the lymphedema QoL decrement was set to zero, the intervention led to a loss in total QALYs (0.4 to 4.0 QALYs per 1000 patients) compared with the standard of care. Assuming a life-long QoL decrement of − 0.013 or more resulted in positive QALY gains in all scenarios, and the NILS model was dominant.

**Table 1:**
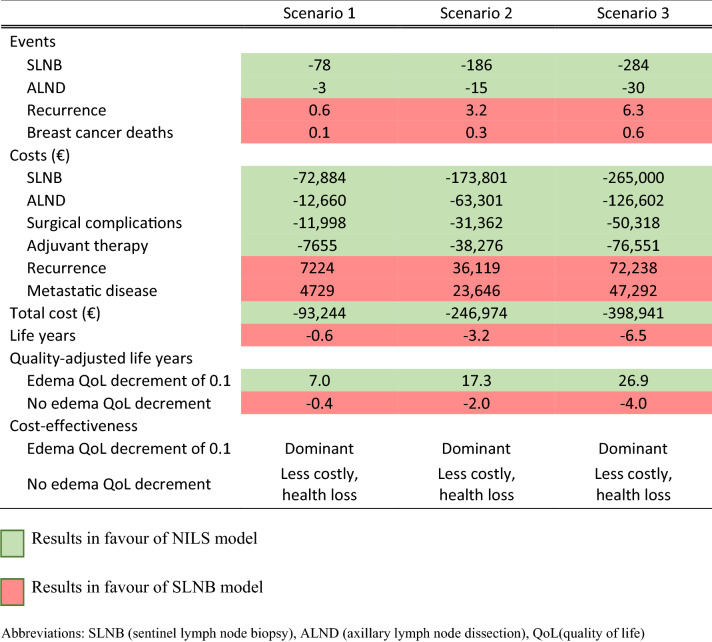
Scenario 1–3 results: Summarized incremental results (in a cohort of *N* = 1000)

**Table 2:**
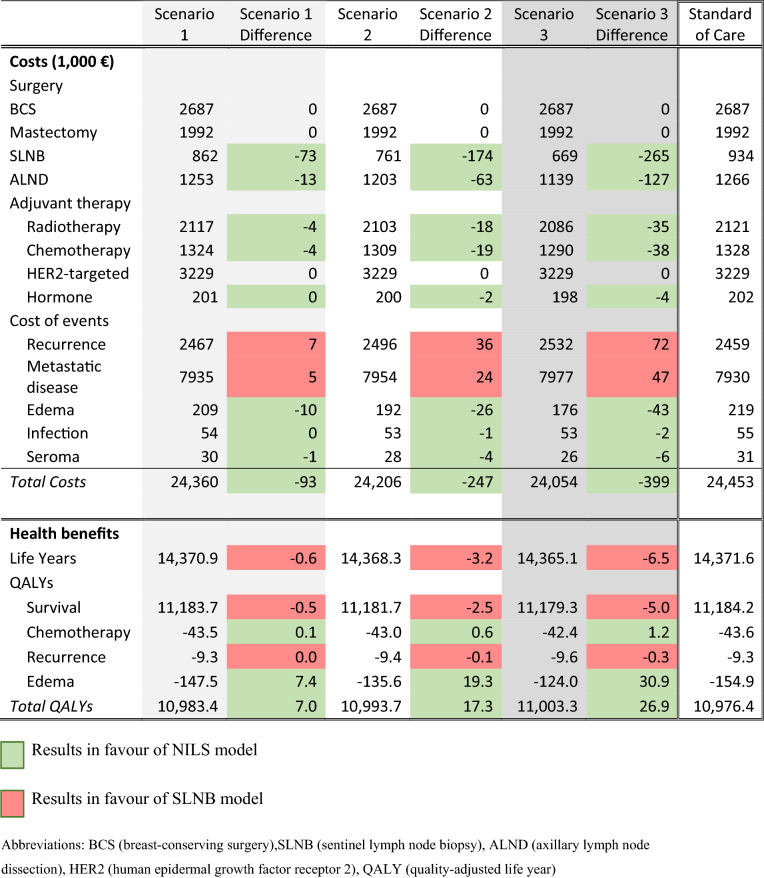
Scenario 1–3 results: Costs (thousands of euros), life years and QALYs (in a cohort of *N* = 1000)

The subgroup analyses showed that implementing the NILS model may be particularly cost effective in patients undergoing BCS (Table 3).

**Table 3:**
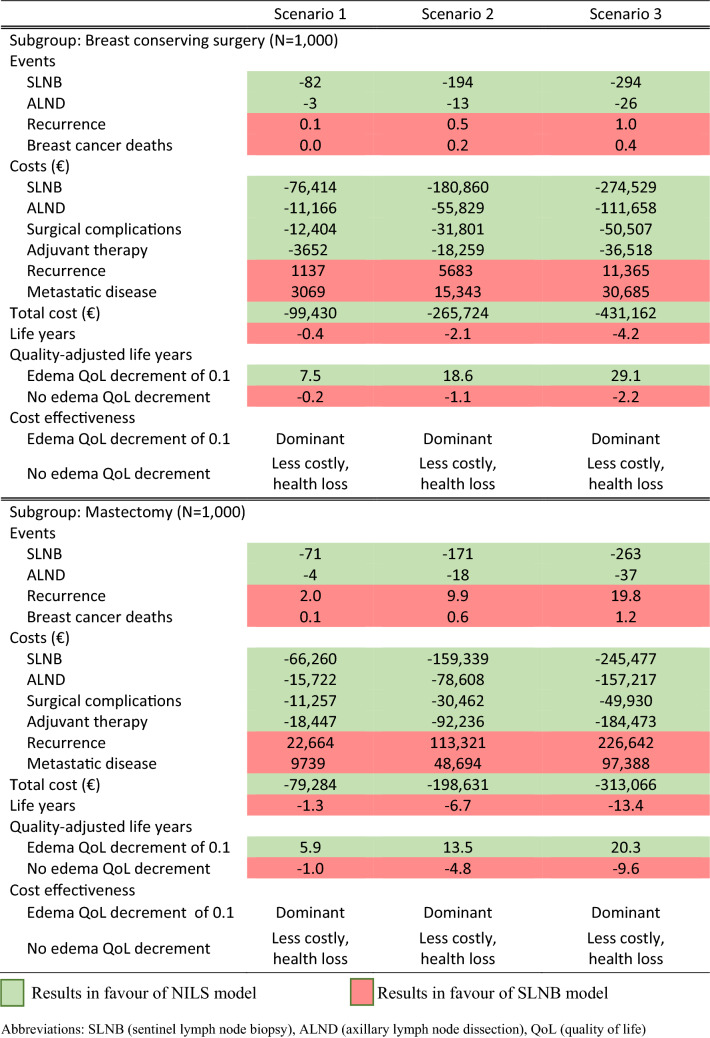
Scenario 1–3 results: Summarized incremental results (in a cohort of *N* = 1000); subgroups were based on breast surgery procedure (breast-conserving surgery *N* = 1000 and mastectomy *N* = 1000)

## Discussion

The NILS model evaluated in this study was developed to predict ALN status with the aim of reducing SLNB in patients with a low predicted risk of nodal metastasis. Limiting the number of patients undergoing surgery could reduce health care costs and lead to gains in quality of life through a reduction in cases of lymphedema. Our analyses describe the impact on health benefits and costs of using the NILS model under different plausible scenarios. In a cohort of 1,000 patients, the health economic model predicted overall benefits of using the NILS model compared to standard of care with cost savings in the range € 93,000 to € 399,000 and a health gain in the range of 7–27 QALYs, depending on levels of sensitivity and specificity of the NILS model (Table 2).

Using the NILS model to guide SLNB decisions would particularly benefit patients undergoing BCS due to the low expected mortality in this subgroup. Additionally, there was a low expected impact of false negative nodal prediction in this patient group since external breast irradiation was generally performed regardless of ALN status. In addition, according to recurrence data from the ACOSOG Z0011 trial [8], it can be expected that while irradiating the breast, the lower part of the axilla will also receive radiation. The economic evaluation assumed that false negative guidance from the NILS model would not alter the clinical decision to give adjuvant radiotherapy in the BCS subgroup, an assumption not applicable to patients undergoing mastectomy. Our results are therefore of clinical importance, showing that the omission of SLNB is also most cost effective in this subgroup. In patients undergoing diagnostic lumpectomy with a finding of invasive cancer, the NILS model could also be applied postoperatively to assess the probability of non-malignant lymph nodes.

Our results support the validity of ongoing clinical trials (SOUND/INSEMA/BOOG), which will hopefully provide more knowledge regarding the safety of omitting SLNB in early BC with clinical and ultrasound-negative axillary findings [9–11]. Moreover, in the subgroup of patients ≥ 70 years with cN0, HR + , and HER2- BC, the updated Canadian ASCO guidelines recommend omission of SLNB if adjuvant endocrine treatment is prescribed [3, 4]. McEvoy et al. performed cost-effectiveness analyses comparing observation versus SLNB in this particular subgroup of patients (postmenopausal patients with HR + /HER2- T1-T2cN0 BC) [30] and found that observation resulted in lower costs and higher QALYs in patients with N0 or N1 BC. These results are in line with our results, although our study population was not as narrowly defined, and included breast cancer patients of all ages regardless of tumor size and HR/HER2 status.

The Swedish guidelines still recommend SLNB in all cN0 patients, while, for example, the Canadian ASCO guidelines [3] have been updated recently. The consideration of only chronological age, not biological age, might raise concerns for a similar implementation in a European context. Regional data show that 32% of the cohort is estimated to belong to this subgroup (ER + and HER2- disease and age ≥ 70 years) [31], and if the ASCO guidelines would be applied, these patients would not be under consideration for the NILS model. We hypothesize that omitting the subgroup fulfilling the Canadian ASCO guidelines from the analysis will only marginally influence the result of the cost-effectiveness analysis.

Restricting the analysis to patients younger than 70 years might strengthen the results, since younger patients have more life years with lymphedema. This assumes that the expected QoL loss from complications due to surgery outweighs the risk of recurrence and early death due to BC. In a scenario with negligible impact on QoL from complications of surgery, SLNB might be more favorable in younger patients given that they have more time at risk for recurrence and a larger expected number of life years lost in the case of death due to BC.

It is important to consider a patient’s individual preferences and make clinical decisions on a patient-to-patient basis [3]; our NILS model provides an individualized estimated probability of healthy lymph node status. The presented health economic model highlights that there may be a trade-off in health to consider, reduced well-being for women burdened by lymphedema vs. reduced length and quality of life for a smaller number of women with undetected recurring BC.

Our analysis highlights a dilemma in the care of BC, in which alternative strategies are associated with risks of negative consequences. Both SLNB and the NILS model are associated with an observed false-negative rate in the ability to detect nodal pathologies in BC and patients with a low risk of N + disease. The current standard of care provides SLNB to all cN0 patients, and the false-negative rate is generally considered to be approximately 10% [32, 33], ranging from 5 to 23% [34], implying that the SLNB technique is associated with a risk of leaving metastatic nodes behind. Moreover, the risk of lymphedema after SLNB is not negligible (Supplementary Material 1). Hence, women choosing this option are not guaranteed to avoid negative health outcomes. In addition, logistical constraints (availability of radiopharmaceuticals), costs, and surgery resources are allocated to patients in whom benefits are clearly limited, i.e., in patients undergoing BCS. The alternative, to limit the number of patients with identified low risk who undergo SLNB, is associated with a slightly increased risk of undetected N + BC. Our analyses depicted three alternative scenarios and described how this risk may translate to lost life years per 1000 patients from a 10-year perspective. Furthermore, the same group of patients who do not undergo SLNB will not be at risk for lymphedema. In addition, it is likely that undetected ALN metastases identified during observation will be actively and effectively treated at the time of detection, indicating that surveillance merely delays curable treatment.

### Impact of lymphedema on QoL

Previous studies have demonstrated that complications and costs associated with SLNB significantly impact patients’ quality of life [35, 36]; however, few studies have used generic instruments suitable for health economic evaluation. In our study, the impact of lymphedema on quality of life had a central role in the analyses. The NILS model led to a positive gain in total QALYs in all three scenarios if the lymphedema QoL decrement was larger than or equal to 0.013. In other words, the expected QoL decrement from lymphedema could be relatively small, and the health gains from the reduced risk of lymphedema could still outweigh the increased risk of recurrence and mortality.

### Strengths and limitations

We present the results from a model-based study where the model structure and data inputs were reported in a transparent way in the main text with additional information in the supplementary material. Among the strengths of the study is that we explored three different scenarios with alternative thresholds for acceptable sensitivity and specificity in the application of the NILS model. The inclusion of BC patients regardless of subtype is also a strength, as it provides broader generalizability. One limitation of our study is that all patients were assumed to be treated according to standard regimens/clinical guidelines, thereby disregarding patients´ and caregivers´ preferences and deviations from given BC treatment that are present in a real-world setting. However, according to national registers with comprehensive coverage, the majority of patients are treated according to clinical guidelines, and we therefore estimate the influence of treatment deviations, on a group level, to be insignificant. Moreover, for consistency with the first publication of the NILS model [12], we included patients with tumors of all sizes in this study. In the context of ongoing clinical trials that have restricted inclusion to patients with tumors ≤ 2 cm (SOUND) and ≤ 5 cm (INSEMA and BOOG), the broad criteria in our study could be considered a limitation. However, in the original NILS dataset, only 5 of 800 patients had tumors > 5 cm [12], and these large tumors are therefore assumed to be of minor influence on the operation of the NILS model and subsequently the results of this cost-effectiveness analysis.

### Future perspective

The NILS model is currently being validated, and the web interface is being thoroughly evaluated. This health economic study further strengthens the utility of future implementation of the NILS model, and such an analysis was a prerequisite for policy-makers to make informed decisions regarding future implementation.

## Conclusion

Compared to the standard of care of SLNB in all cN0 BC patients, the adoption of an ANN decision tool, such as the NILS model presented here, for predicting ALN status in patients with cN0 BC can reduce health care costs from surgery and drugs and provide gains in quality of life, with particularly pronounced effects in patients undergoing BCS. The NILS model was dominant in terms of costs and QALYs compared with standard of care when the life-long lymphedema QoL decrement was set to 0.1. When excluding the lymphedema QoL decrement, the NILS model resulted in lower total costs and QALYs than standard of care and was most cost effective in the subgroup of patients undergoing BSC. The evaluation of costs and quality-of-life outcomes when omitting SLNB will complement the upcoming results of ongoing clinical trials and provide a foundation for clinical guidelines.

## Supplementary Information

Below is the link to the electronic supplementary material.

Supplementary file1 (DOCX 53 KB)

## Data Availability

The raw datasets are available from the corresponding author on reasonable request due to restrictions, i.e., privacy or ethical restrictions.

## Abbreviations

BC: Breast cancer
ALN: Axillary lymph node
SLN: Sentinel lymph node
SLNB: Sentinel lymph node biopsy
ALND: Axillary lymph node dissection
BCS: Breast-conserving surgery
HR: Hormone receptor
HER2: Human epidermal growth factor receptor 2
ANN: Artificial neural network
NILS: Noninvasive lymph node staging
ER: Estrogen receptor
QALY: Quality-adjusted life year
QoL: Quality of life
ICER: Incremental cost-effectiveness ratio
