# Supplementary material for: The implementation of a noninvasive lymph node staging (NILS) preoperative prediction model is cost effective in primary breast cancer
Source: Breast Cancer Res Treat. 2022 Jul 5;194(3):577–86. doi: 10.1007/s10549-022-06636-x (PMC9287207; doi:10.1007/s10549-022-06636-x)
Supplement: Supplementary file 1 — Supplementary file1 (DOCX 53 KB) [file 10549_2022_6636_MOESM1_ESM.docx]

Supplementary Material 1. Input parameters used in the model simulation: probabilities and costs

| Variable type | Variable description | Assumed value | Reference |
| --- | --- | --- | --- |
| Population | Number of included patients (N) | 1,000 | Assumption |
|  | Median age (years) | 66 | [1] |
|  | Proportion of pathological nodal disease in clinically node negative BC | 32% | [2] |
| Test accuracy (probabilities) | SLNB (sensitivity: correct finding N+, i.e. N+ versus N0) | 92.3% sensitivity and 100% specificity | [3] |
|  | ALND (N+ versus N0) | 100% sensitivity and specificity | Assumption |
|  | The NILS model (N0 versus N+) | Scenario 1: 99% sensitivity and 11% specificity  Scenario 2: 95% sensitivity and 25% specificity  Scenario 3: 90% sensitivity and 37% specificity | [4] |
| Treatments (probabilities) | Proportion of BCS | 67.2% | [1] |
|  | Radiotherapy after BCS | 94.3%, 15 fractions | [1] |
|  | Radiotherapy after mastectomy with pathological node positive BC | 82.7%, 25 fractions | [1] |
|  | Chemotherapy (3 x EC + 3 x Docetaxel) - pathological nodal **negative** BC | 26% | [5] |
|  | Chemotherapy (3 x EC + 3 x Docetaxel) - pathological nodal **positive** BC | 61% | [5] |
|  | Endocrine treatment ER+ BC  [37% tamoxifen and 63% aromatase inhibitors (letrozole)] | 89.3%  Nodal negative: 5 years  Nodal positive: 10 years | [6,1,7] |
|  | HER2-targeted therapy (Trastuzumab) – HER2 positive BC (pathological node positive and negative) | 64.1% | [1] |
| Complications (probabilities) | Lymphedema without axillary surgery | 0.4% | [8] |
|  | Lymphedema after SLNB | 6.3% | [9] |
|  | Lymphedema after SLNB and ALND | 22.3% | [9] |
|  | Seroma without axillary surgery | 3.2% | [8] |
|  | Seroma after SLNB | 11% | [10] |
|  | Seroma after SLNB and ALND | 20% | [10] |
|  | Infection without axillary surgery | 9.8% | [8] |
|  | Infection after SLNB | 11% | [11] |
|  | Infection after SLNB and ALND | 15% | [11] |
| Health effects (Annual probabilities) | Yearly mortality rate - pathological nodal **negative** BC | 0.7% | [12] |
|  | Yearly mortality rate - pathological nodal **positive** BC | 1.8% | [12] |
|  | Yearly recurrence rate - pathological nodal **negative** BC | 1.8% | [12] |
|  | Yearly recurrence rate - pathological nodal **positive** BC | 3.8% | [12] |
| Subgroup analysis | Hazard ratio mortality mastectomy vs. BCS - pathological nodal **negative** BC | 1.76/1.1 | [12] |
|  | Hazard ratio mortality mastectomy vs. BCS - pathological nodal **positive** BC | 1.36/1 | [12] |
|  | Proportion of pathological nodal disease - mastectomy subgroup | 40% | [2] [12] |
|  | Proportion of pathological nodal disease - BCS subgroup | 29% | [2] [12] |
| Treatment effects (pN+ BC) (annual probabilities) | Yearly risk ratio of survival from omitting radiotherapy after mastectomy (mastectomy + radiotherapy vs mastectomy alone) | 0.92 | [13] |
|  | Yearly risk ratio of survival from omitting chemotherapy (anthracycline-based chemotherapy vs. no chemotherapy) | 0.79 | [14] |
|  | Yearly risk ratio of survival from omitting an additional five years of hormonal treatment (HR+ BC) | 0.985  Mean value of 1 (no effect, reflecting the first 5 years of hormone treatment) and 0.97 (reflecting the effects from lengthened therapy, year 5-9). | [15] |
|  | Yearly risk ratio of recurrence from omitting radiotherapy after mastectomy (mastectomy + radiotherapy vs mastectomy alone) | 0.34 | [13] |
|  | Yearly risk ratio of recurrence from omitting chemotherapy (anthracycline-based chemotherapy vs no chemotherapy) | 0.79 | [14] |
|  | The yearly risk ratio of survival from omitting an additional five years of hormonal treatment (HR+ BC) | 0.95  Mean value of 1 (no effect, reflecting the first 5 years of hormone treatment) and 0.90 (reflecting the effects from lengthened therapy, year 5-9). | [15] |
| COSTS (EUR, year 2020 price level) | | | |
| Surgical procedures | BCS | 3,999 | Skåne University Hospital |
|  | Mastectomy | 6,074 | Skåne University Hospital |
|  | SLNB | 934 | Skåne University Hospital |
|  | ALND | 4,286 | Skåne University Hospital |
| Adjuvant therapy | Radiotherapy preparation (one-time cost) | 391 | CPP database |
|  | Radiotherapy (per visit) | 160 | CPP database |
|  | Chemotherapy drug cost (six cycles) | 1,883 | TLV database, pharmacy price lists |
|  | Chemotherapy administration (per visit) | 295 | Southern Healthcare Region Pricelist 2020 |
|  | Endocrine therapy drug cost (1 year) | 54 | TLV database, Swedish Prescribed Drug Register |
|  | HER2-targeted therapy drug cost (1 year) | 28,984 | Pharmacy price lists |
| Complications | Lymphedema (one-time cost) | 1,986  Six oncology visits (EUR 517 for the first visit and EUR 258 for each re-visits) plus the cost of one compression sleeve kit (EUR 228). | Southern Healthcare Region Pricelist 2020, pharmacy price lists |
|  | Infection (one-time cost) | 448  One physician visit (EUR 220) and two nurse visits (EUR 114 for each visit). | Southern Healthcare Region Pricelist 2020 |
|  | Seroma (one-time cost) | 228  Two nurse visits (EUR 114 for each visit). | Southern Healthcare Region Pricelist 2020 |
| Recurrence | Recurrence | 13,231 | [16] |
| Metastatic disease | Metastatic disease | 96,535 | [17] |
| QUALITY OF LIFE (EQ-5D) | | | |
|  | Adjuvant chemotherapy  (initial treatment, one year) | -0.12 | [18,19] |
|  | Adjuvant radiotherapy and endocrine therapy | No data |  |
|  | Recurrence  (one year) | -0.05  Average between  local (0) and distant recurrence (-0.10) | [18,19] |
|  | Lymphedema  (lifelong) | With and without lymphedema disutility | [20,21] |

Abbreviations: ALND: axillary lymph node dissection; BC: breast cancer; BCS: breast conserving surgery; EC: epirubicin and cyclophosphamide; HER2: human epidermal receptor 2; HR: hormone receptor; NILS: non invasive lymph node staging; SLNB: sentinel lymph node biopsy

Reference list:

1. Yearly report 2019 (interactive) (2020). Accessed Nov 2020

2. Majid S, Tengrup I, Manjer J (2013) Clinical assessment of axillary lymph nodes and tumor size in breast cancer compared with histopathological examination: a population-based analysis of 2,537 women. World J Surg 37 (1):67-71. doi:10.1007/s00268-012-1788-5

3. Bergkvist L, Frisell J, Swedish Breast Cancer G, Swedish Society of Breast S (2005) Multicentre validation study of sentinel node biopsy for staging in breast cancer. Br J Surg 92 (10):1221-1224. doi:10.1002/bjs.5052

4. Dihge L, Ohlsson M, Eden P, Bendahl PO, Ryden L (2019) Artificial neural network models to predict nodal status in clinically node-negative breast cancer. BMC Cancer 19 (1):610. doi:10.1186/s12885-019-5827-6

5. (NKBC) NQRfBC (2016) Yearly rapport 2015. <https://cancercentrum.se/globalassets/cancerdiagnoser/brost/kvalitetsregister/nationell_brostcancer_rapport_2015_revc.pdf>

6. Group SBC (2020) Bröstcancer - nationellt vårdprogram (Clinical Practice Guidelines on Breast Cancer) <https://kunskapsbanken.cancercentrum.se/globalassets/cancerdiagnoser/brost/vardprogram/nationellt-vardprogram-brostcancer.pdf>

7. Welfare NBoHa (2021) The Swedish Prescribed Drug Register. <https://sdb.socialstyrelsen.se/if_lak/val.aspx>. Accessed 23 September 2021

8. Killelea BK, Long JB, Dang W, Mougalian SS, Evans SB, Gross CP, Wang SY (2018) Associations Between Sentinel Lymph Node Biopsy and Complications for Patients with Ductal Carcinoma In Situ. Ann Surg Oncol 25 (6):1521-1529. doi:10.1245/s10434-018-6410-0

9. Shaitelman SF, Cromwell KD, Rasmussen JC, Stout NL, Armer JM, Lasinski BB, Cormier JN (2015) Recent progress in the treatment and prevention of cancer-related lymphedema. CA Cancer J Clin 65 (1):55-81. doi:10.3322/caac.21253

10. Purushotham AD, Upponi S, Klevesath MB, Bobrow L, Millar K, Myles JP, Duffy SW (2005) Morbidity after sentinel lymph node biopsy in primary breast cancer: results from a randomized controlled trial. J Clin Oncol 23 (19):4312-4321. doi:10.1200/JCO.2005.03.228

11. Mansel RE, Fallowfield L, Kissin M, Goyal A, Newcombe RG, Dixon JM, Yiangou C, Horgan K, Bundred N, Monypenny I, England D, Sibbering M, Abdullah TI, Barr L, Chetty U, Sinnett DH, Fleissig A, Clarke D, Ell PJ (2006) Randomized multicenter trial of sentinel node biopsy versus standard axillary treatment in operable breast cancer: the ALMANAC Trial. J Natl Cancer Inst 98 (9):599-609. doi:10.1093/jnci/djj158

12. Andersson Y, Bergkvist L, Frisell J, de Boniface J (2018) Long-term breast cancer survival in relation to the metastatic tumor burden in axillary lymph nodes. Breast Cancer Res Treat 171 (2):359-369. doi:10.1007/s10549-018-4820-0

13. Clarke M, Collins R, Darby S, Davies C, Elphinstone P, Evans V, Godwin J, Gray R, Hicks C, James S, MacKinnon E, McGale P, McHugh T, Peto R, Taylor C, Wang Y, Early Breast Cancer Trialists' Collaborative G (2005) Effects of radiotherapy and of differences in the extent of surgery for early breast cancer on local recurrence and 15-year survival: an overview of the randomised trials. Lancet 366 (9503):2087-2106. doi:10.1016/S0140-6736(05)67887-7

14. Early Breast Cancer Trialists' Collaborative G, Peto R, Davies C, Godwin J, Gray R, Pan HC, Clarke M, Cutter D, Darby S, McGale P, Taylor C, Wang YC, Bergh J, Di Leo A, Albain K, Swain S, Piccart M, Pritchard K (2012) Comparisons between different polychemotherapy regimens for early breast cancer: meta-analyses of long-term outcome among 100,000 women in 123 randomised trials. Lancet 379 (9814):432-444. doi:10.1016/S0140-6736(11)61625-5

15. Davies C, Pan H, Godwin J, Gray R, Arriagada R, Raina V, Abraham M, Medeiros Alencar VH, Badran A, Bonfill X, Bradbury J, Clarke M, Collins R, Davis SR, Delmestri A, Forbes JF, Haddad P, Hou MF, Inbar M, Khaled H, Kielanowska J, Kwan WH, Mathew BS, Mittra I, Muller B, Nicolucci A, Peralta O, Pernas F, Petruzelka L, Pienkowski T, Radhika R, Rajan B, Rubach MT, Tort S, Urrutia G, Valentini M, Wang Y, Peto R, Adjuvant Tamoxifen: Longer Against Shorter Collaborative G (2013) Long-term effects of continuing adjuvant tamoxifen to 10 years versus stopping at 5 years after diagnosis of oestrogen receptor-positive breast cancer: ATLAS, a randomised trial. Lancet 381 (9869):805-816. doi:10.1016/S0140-6736(12)61963-1

16. Lidgren M, Wilking N, Jonsson B, Rehnberg C (2007) Resource use and costs associated with different states of breast cancer. Int J Technol Assess Health Care 23 (2):223-231. doi:10.1017/S0266462307070328

17. Dahlberg L, Lundkvist J, Lindman H (2009) Health care costs for treatment of disseminated breast cancer. Eur J Cancer 45 (11):1987-1991. doi:10.1016/j.ejca.2009.03.023

18. Lidgren M, Wilking N, Jonsson B, Rehnberg C (2007) Health related quality of life in different states of breast cancer. Qual Life Res 16 (6):1073-1081. doi:10.1007/s11136-007-9202-8

19. Hall PS, McCabe C, Stein RC, Cameron D (2012) Economic evaluation of genomic test-directed chemotherapy for early-stage lymph node-positive breast cancer. J Natl Cancer Inst 104 (1):56-66. doi:10.1093/jnci/djr484

20. Herberger K, Blome C, Heyer K, Ellis F, Munter KC, Augustin M (2017) Quality of life in patients with primary and secondary lymphedema in the community. Wound Repair Regen 25 (3):466-473. doi:10.1111/wrr.12529

21. Cheville AL, Almoza M, Courmier JN, Basford JR (2010) A prospective cohort study defining utilities using time trade-offs and the Euroqol-5D to assess the impact of cancer-related lymphedema. Cancer 116 (15):3722-3731. doi:10.1002/cncr.25068
